# Nicardipine sensitizes temozolomide by inhibiting autophagy and promoting cell apoptosis in glioma stem cells

**DOI:** 10.18632/aging.202539

**Published:** 2021-02-17

**Authors:** Jia Shi, Xuchen Dong, Haoran Li, Haiyang Wang, Qianqian Jiang, Liang Liu, Liping Wang, Jun Dong

**Affiliations:** 1 Department of Neurosurgery, Second Affiliated Hospital of Soochow University, Suzhou, China; 2 Department of Neurosurgery, Third Affiliated Hospital of Soochow University, Changzhou, China

**Keywords:** nicardipine, apoptosis, autophagy, glioblastoma multiforme, glioma stem cells

## Abstract

Glioblastoma multiforme (GBM) is the most invasive malignant central nervous system tumor with poor prognosis. Nicardipine, a dihydropyridine calcium channel antagonist, has been used as an adjuvant to enhance sensitivity to chemotherapeutic drugs. However, whether glioma stem cells (GSCs) can be sensitized to chemotherapy via combined treatment with temozolomide (TMZ) and nicardipine is unclear. In this study, surgical specimen derived GSCs SU4 and SU5 were applied to explore the sensitization effect of nicardipine on temozolomide against GSCs, and further explore the relevant molecular mechanisms. Our results showed that nicardipine can enhance the toxic effect of temozolomide against GSCs, promote apoptosis of GSCs, and inhibit autophagy of GSCs. The relevant mechanisms were related to activation of mTOR, and selective inhibition of mTOR by rapamycin could weaken the sensitization of nicardipine to temozolomide, which suggest that nicardipine can be applied as an adjuvant to inhibit autophagy in GSCs, and enhance apoptosis-promoting effect of temozolomide in GSCs as well. Nicardipine can inhibit autophagy by activating expression of mTOR, thus play tumor inhibition roles both *in vitro* and *in vivo*. Repurposing of nicardipine can help to improving therapeutic effect of TMZ against GBM, which deserves further clinical investigations.

## INTRODUCTION

Glioblastoma multiforme (GBM), as WHO IV grade intracranial tumor, is the most common and invasive primary adult brain tumor, accounting for 46.6% of glioma cases [1]. Chemotherapy is one of the most important therapeutic procedure against GBM. Alkylating agents, including TMZ and carmustine, have been widely administered as post-operation treatment against GBM [2–4]. However, despite certain advances in chemotherapy, the outcome of GBM has not been improved obviously, with comprehensive therapies resulting in a median overall survival time of approximately 15-18 months [3] and a 5-year survival rate of only 5.5% [1]. Based on recent studies on GSCs [5] and their microenvironment [6], GSCs are considered the main driver of GBM tissue remodeling, which leads to tumor heterogeneity and chemotherapeutic resistance. Further exploration of effective molecular targets against GSCs in order to develop new therapeutics is on urgent need to improve outcome of GBM patients.

Drug repurposing to play synergistic roles with the classical chemotherapeutic drugs have become novel potential strategies in development of new treatment regimen [7, 8]. Great progress has recently been made in repurposing of the traditional non-cancer drugs, for example, metformin and sildenafil, as alternative therapeutics for cancer treatment, which may produce great social and economic benefits in reducing costs in drug research and development [9–11]. Nicardipine, acts as a dihydropyridine calcium channel antagonist effecting on vascular smooth muscle and reducing blood pressure, is commonly used for blood pressure control in stroke patients [12, 13]. However, the potential chemotherapeutic enhancing effect of Nicardipine on TMZ against GBM has not been fully elucidated [12–14].

Similar to other calcium channel blockers, Nicardipine acts by inhibiting the influx of calcium ions into vascular smooth muscle and cardiac cells during depolarization. Blockade of calcium flux leads to vasodilation and a decrease in cardiac load and oxygen consumption [15]. To date, several studies have demonstrated that the pharmacological effects of nicardipine vary among different kinds of cells and tissues. Apoptotic cell death was observed in nicardipine-exposed human microvascular endothelial cells [16] and rat cerebellar granule neurons [17]. Autophagy occurs under different types of physiological or pathological stimulation [18]. During autophagy, cells degrade damaged, misfolded to form dysfunctional proteins via the lysosomal pathway [19]. Current studies show that dysregulation of autophagy may induce inverse effects on cancer therapy depending on different cellular environment [8, 20]. Therefore, whether autophagy is involved in the chemotherapeutic enhancing effects of nicardipine and whether nicardipine can synergize with the first line chemotherapeutic drugs, TMZ against gliomas need to be clarified.

In the current studies, we explored the role of nicardipine in GSCs autophagy and sensitization of TMZ against GSCs, the relevant potential molecular mechanisms have been investigated as well, for the purpose of evaluating whether nicardipine can be a potential adjuvant for postoperative chemotherapy against GSCs, and a part of the comprehensive treatment approach to enhance the effect of chemotherapy against GBM.

## RESULTS

### Susceptibility of GSCs to combining administering of TMZ and nicardipine

**Figure 1:**
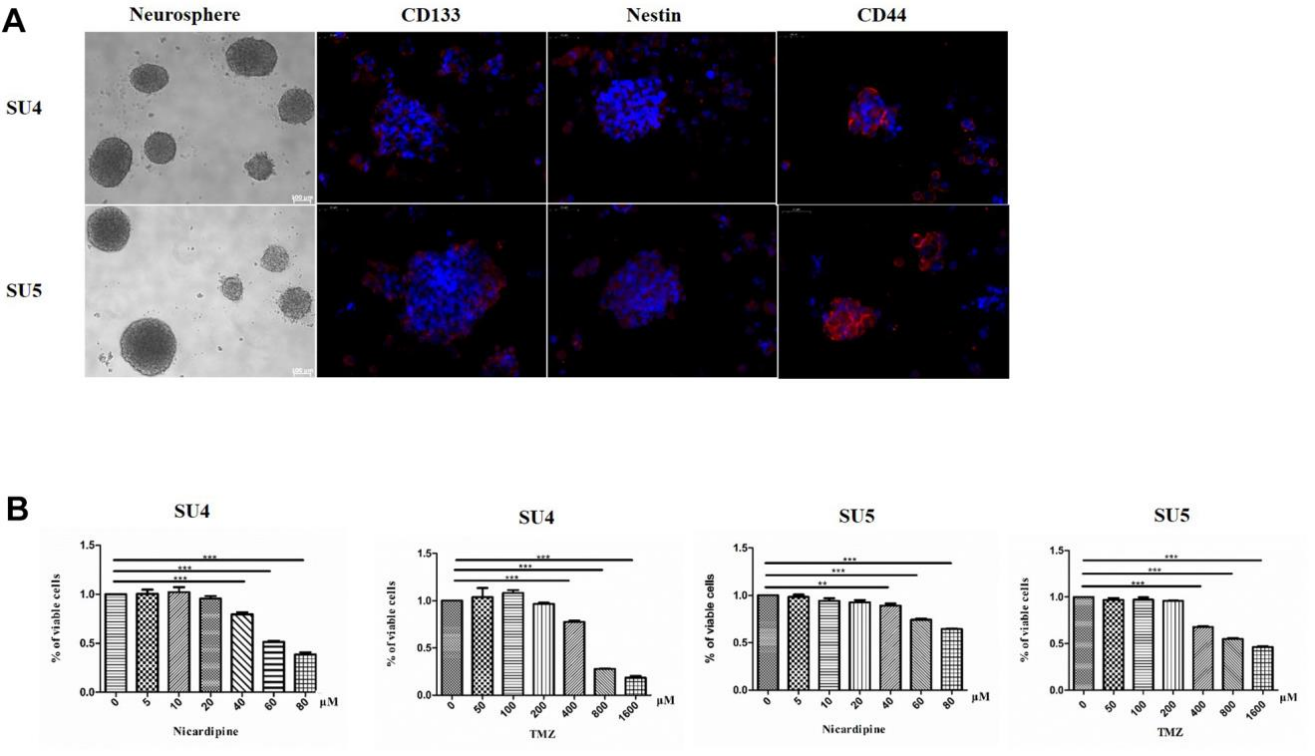
**GSCs characteristic analysis and cell survival assay evaluating GSCs sensitivity to TMZ and nicardipine.** Under the phase contrast microscope, GSCs showed spherical growth (Scale bar 100μm); CD133, Nestin, and CD44 were positively expressed in GSCs, determined by immunofluorescence staining (Scale bar 50μm) (**A**). Cell survival assay by CCK-8 after administering TMZ ranging from 0 to 1600 μM, nicardipine (0 to 80 μM), or both for 48 h (**B**). ** *p* < 0.01, *** *p* < 0.001.

SU4 and SU5 cells derived from surgical specimen of glioblastoma patients were applied for cytotoxicity experiments. GSCs (both SU4 and SU5) had a strong ability to form tumor-sphere like cell clusters, and expressed GSCs markers, including CD133, nestin and CD44, consistent with the GSCs stemness characteristics (Figure 1A). To evaluate the cytotoxic effect of TMZ and nicardipine on GSCs, both SU4 and Su5 cells were incubated at the indicated concentrations of TMZ or nicardipine for 48 h. Cell viability was detected by CCK-8 assay, which showed that GSCs were highly resistant to TMZ at the regular concentration, decrease in cell viability appeared at TMZ concentration higher than 400 μM (Figure 1B). Inhibitory effect of TMZ on GSCs at lower concentration (100μm) was poor even after longer time point (96 h) (Supplementary Figure 1). This was related to the innate tolerance of GSCs to chemotherapy [21, 22]. Nicardipine showed no obvious cytotoxic effects against GSCs at the low concentration (<40 μM) (Figure 1B). In order to avoid confusion caused by the direct inhibition effect of high concentration of nicardipine on GSCs, we selected low concentration nicardipine (20 μM) as chemosensitizer. Combined with the preliminary experimental data results and the previous studies [23, 24], 400 μM TMZ and 20 μM nicardipine were selected to explore the sensitization effect of nicardipine on TMZ against GSCs.

### Nicardipine can enhance TMZ cytotoxic effects on GSCs

**Figure 2:**
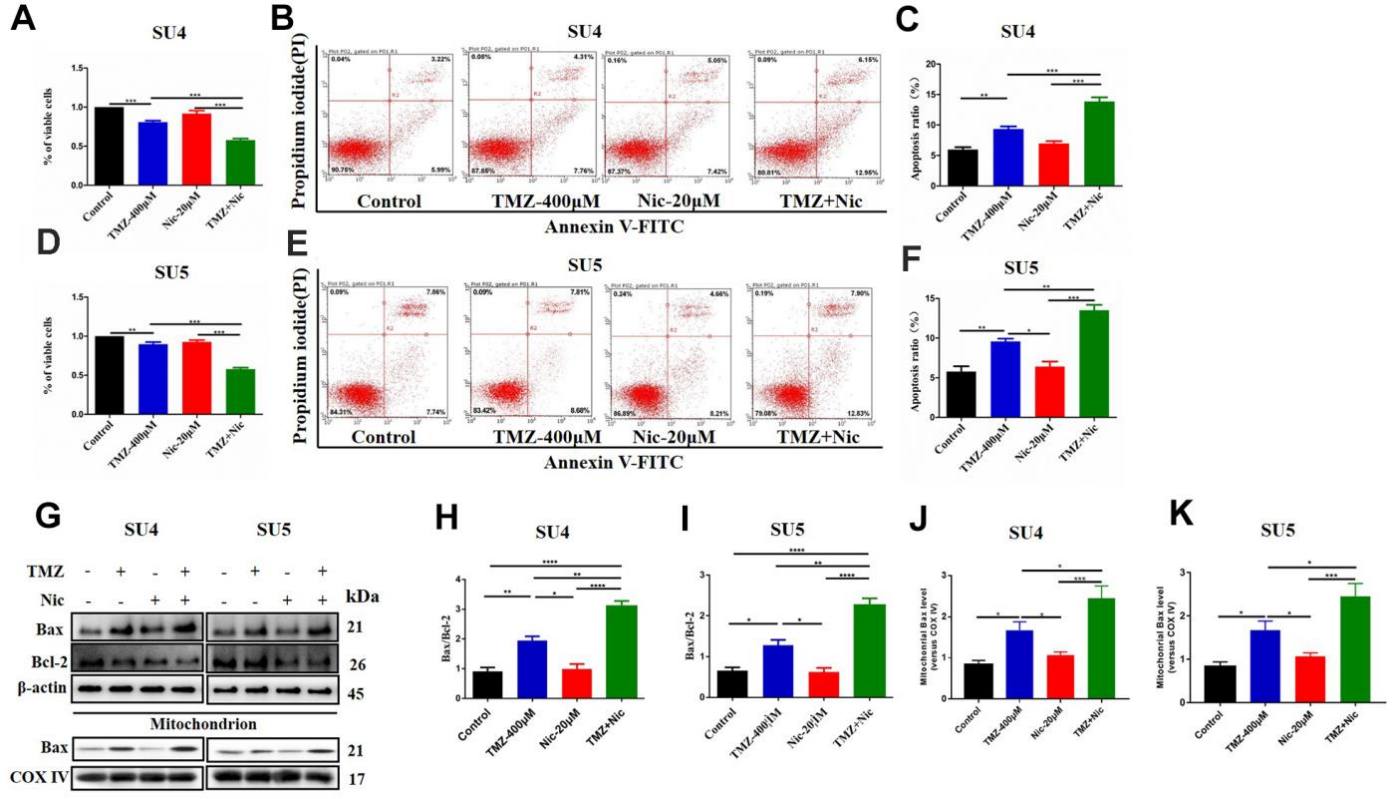
**Combining dosing of nicardipine and TMZ decreased cell viability and promoted apoptosis of GSCs.** SU4 cell viability was assessed by CCK-8 assay under exposure to TMZ (400 μM), nicardipine (20 μM) or both for 48 h (**A**). SU4 cells were treated with TMZ (400 μM), nicardipine (20 μM) or both for 48 h, then were stained with PI and Annexin V-FITC for apoptosis analysis (**B**, **C**). SU5 cell viability was assessed by CCK-8 assay under treatment with TMZ (400 μM), nicardipine (20 μM) or both for 48 h (**D**). SU5 cells were treated with TMZ (400 μM), nicardipine (20 μM) or both for 48 h, then were stained with PI and Annexin V-FITC for apoptosis analysis (**E**, **F**). Western blot analysis of Bcl-2, Bax, mitochondrial Bax and COX IV in GSCs was performed after treatment with TMZ, nicardipine or both for 48 h (**G**–**K**). * *p* < 0.05, ** *p* < 0.01, *** *p* < 0.001, **** *p* < 0.0001.

CCK-8 assay showed that 400 μM TMZ in combination with 20 μM nicardipine obviously reduced the viability of both SU4 and SU5 GSCs (Figure 2A, 2D). Flow cytometry was carried out to determine whether apoptosis of GSCs was induced by combining administration of TMZ and nicardipine. The apoptosis ratio induced by TMZ plus nicardipine was markedly higher than that of TMZ alone (Figure 2B, 2C, 2E, 2F). Several proteins involved in apoptosis-related signaling pathway were detected by Western blot to further verify initiation of apoptosis induced by TMZ in combination with nicardipine, which showed combination of TMZ and nicardipine promoted Bax accumulation obviously (Figure 2G–2I). In addition, mitochondrial proteins of GSCs were extracted to analyze Bax expression in mitochondrion, compared to TMZ group, combination of TMZ and nicardipine can upregulate mitochondrial Bax expression both in SU4 and SU5 cells, confirming activation of mitochondrial apoptosis induced by TMZ plus nicardipine (Figure 2G, 2J, 2K). Taken together, these data suggest that combination of TMZ with nicardipine can inhibit GSCs proliferation via promoting mitochondrial apoptosis.

### Combination of nicardipine and TMZ can inhibit GSCs autophagy via upregulating mTOR

**Figure 3:**
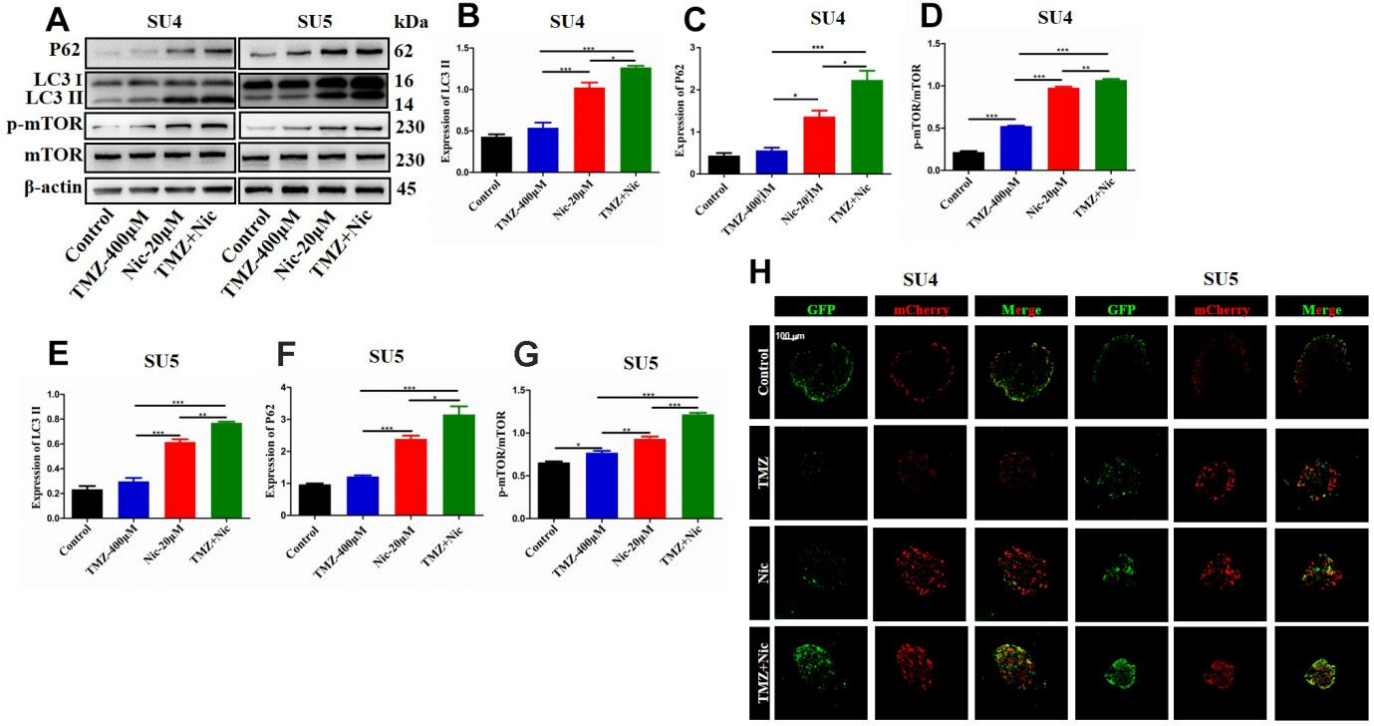
**Autophagy-related proteins expression in GSCs after treating with TMZ, nicardipine, or both.** Western blot analysis of p62, LC3 II and mTOR in SU4 and SU5 cells treated with TMZ (400 μM), nicardipine (20 μM), or both, respectively for 48 h (**A**). Statistical analysis of p62, LC3 II and mTOR protein levels in SU4 and SU5 cells (**B**–**G**). The mCherry-GFP-LC3 distribution in GSCs cultured with TMZ, nicardipine or both after 48 h was analyzed by confocal microscopy. Scale bar 100 μM (**H**). * *p* <0.05, ** *p* <0.01, *** *p* <0.001.

To analyze the effect of TMZ in combination with nicardipine on autophagy of GSCs, the expression of p62 and LC3 in SU4 and SU5 cells were measured. Western blot data showed upregulation of p62 and LC3 expression both in nicardipine group and TMZ plus nicardipine group, p62 and LC3 expression in TMZ plus nicardipine group even higher (*p*<0.05) (Figure 3A–3C, 3E, 3F), suggesting that nicardipine can inhibit both autophagosome transport and autophagy in GSCs [25]. For further verifying this result, LC3 was transfected into GSCs with lentivirus vector to detect autophagic flux via mCherry/GFP dual-color tracing techniques, in which GFP can be quenched in the acidic environment of lysosome, while mCherry emitting red fluorescence can exist longer. The yellow fluorescence formed by fusion of GFP and mCherry represented autophagosome, and the red fluorescence implied the combination of autophagosome and lysosome to form autolysosome. Aggregation of yellow fluorescence in GSCs can be observed treated with TMZ plus nicardipine, which was significantly higher than GSCs treated with TMZ alone, or nicardipine alone, indicating that transportation of autophagy bodies and binding of lysosomes in GSCs decreased obviously, and inhibition of autophagy existed (Figure 3H).

**Figure 4:**
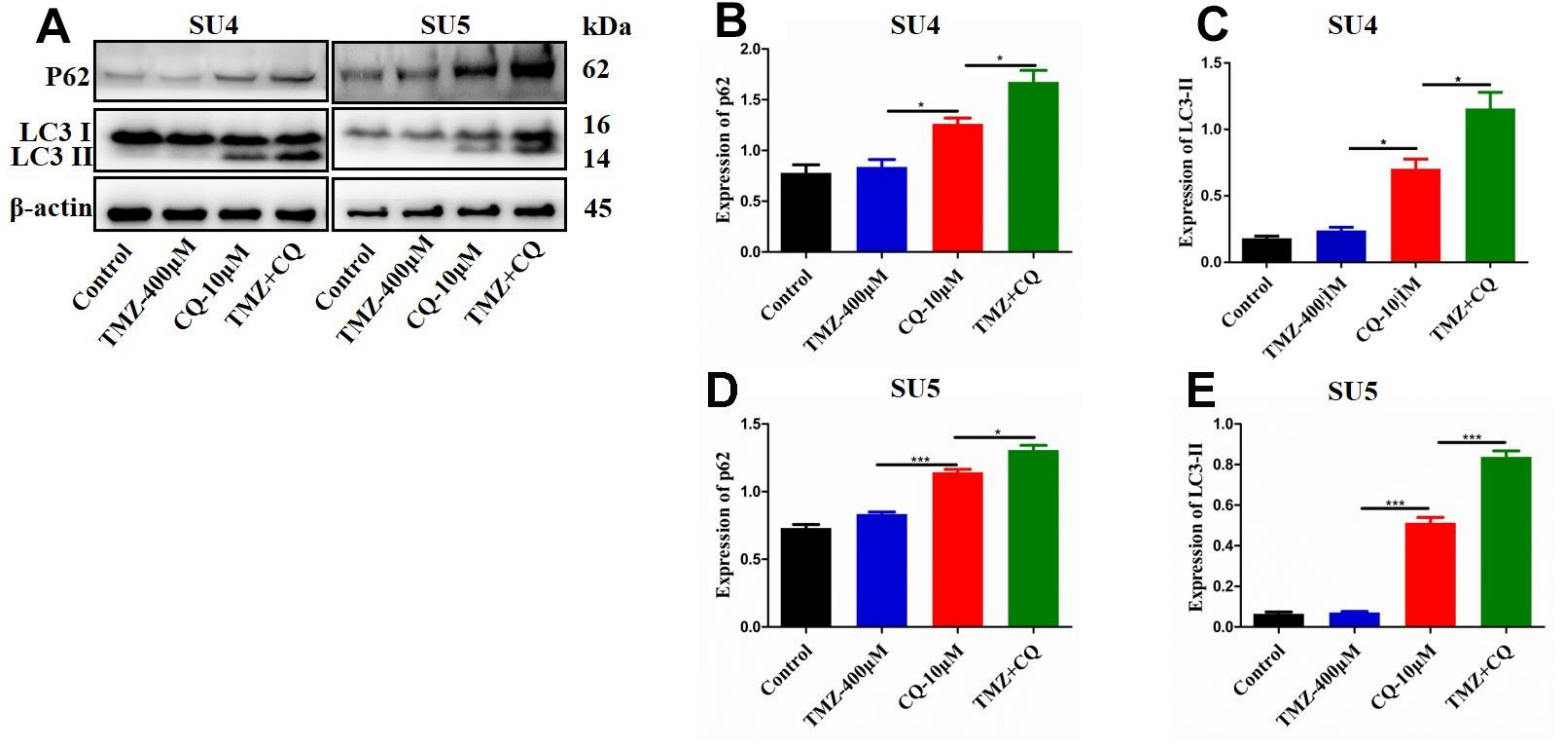
**Detection of autophagy level of GSCs treated with TMZ, CQ, or both.** SU4 and SU5 cells were exposed to TMZ (400 μM), CQ (10 μM), or both, respectively for 48 h. Then p62 and LC3 II expression levels were analyzed by Western blotting (**A**). Statistical analysis of p62 and LC3 II expression in SU4 and SU5 cells (**B**–**E**). *p < 0.05, ***p<0.001.

Chloroquine (CQ), as an inhibitor of late autophagy, was applied to verify the inhibitory role of nicardipine on GSCs autophagy. The combination of TMZ (400 μM) and CQ (10 μM) increased in p62 and LC3-II expression levels in both SU4 and SU5 cells (Figure 4), which accorded with the effect of nicardipine on GSCs. Previous studies reported that mTOR played a key role in autophagy regulation and can inhibit autophagy through multiple molecular regulatory steps [26], then the protein levels of mTOR and phosphorylated-mTOR (p-mTOR) were analyzed in GSCs treated with TMZ and nicardipine, which suggested that the p-mTOR/mTOR ratio exhibited significant increase in GSCs treated with TMZ plus nicardipine (Figure 3A, 3D, 3G), which implied that combining application of nicardipine and TMZ can inhibit GSCs autophagy by upregulating mTOR expression.

### Nicardipine can sensitize TMZ against GSCs by promoting cellular apoptosis via suppressing autophagy

**Figure 5:**
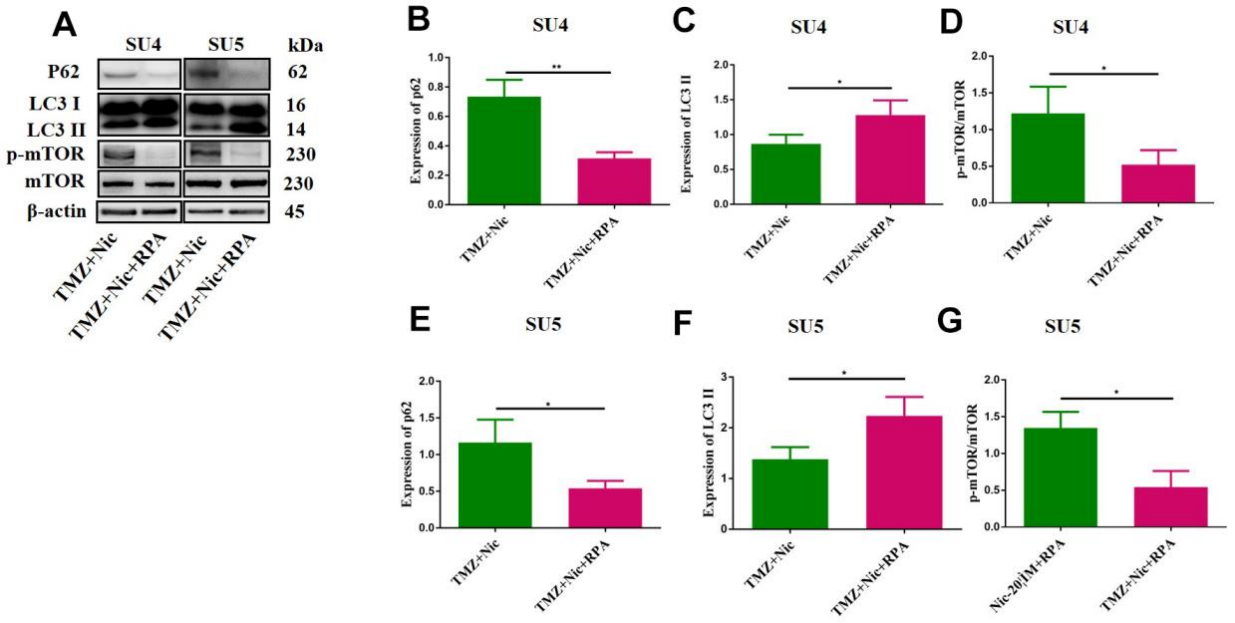
**Rapamycin promoted GSCs autophagy by activating mTOR.** The protein levels of p62, LC3 II and mTOR in GSCs were measured by Western blotting after exposure to TMZ (400 μM) plus nicardipine (20 μM) for 48 h, or in GSCs pretreated with rapamycin (50 nM) for 12 h (**A**). Statistical analysis of p62, LC3 II and mTOR expression in SU4 and SU5 cells (**B**–**G**). *p < 0.05, **p<0.01.

**Figure 6:**
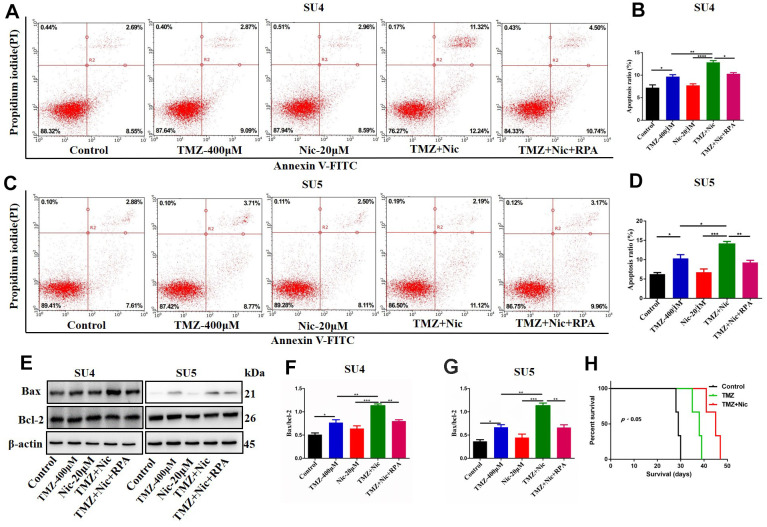
**Rapamycin inhibited nicardipine-induced sensitization of TMZ against GSCs**. SU4 and SU5 cells were treated with TMZ (400 μM), nicardipine (20 μM), TMZ (400 μM) + nicardipine (20 μM), and TMZ (400 μM) + nicardipine (20 μM) +rapamycin (RPA) (50 nM), respectively, for 48 h. Then cells were stained with PI and Annexin V-FITC for apoptosis analysis (**A**–**D**). Western blot analysis of Bcl-2 and Bax in GSCs was performed after treating with TMZ (400 μM), nicardipine (20 μM), TMZ (400 μM) + nicardipine (20 μM), and TMZ (400 μM) + nicardipine (20 μM) +RPA (50 nM), respectively for 48 h (**E**–**G**). The survival analysis of GSCs bearing mice after chemotherapy, Kaplan-Meier curve indicated combined treatment with TMZ and nicardipine had positive survival benefit (**H**). * *p* < 0.05, ** *p* < 0.01, ***p<0.001.

Combination of TMZ and nicardipine increased p-mTOR, p62 and LC3-II expression levels of GSCs (Figure 3). Rapamycin, as a selective mTOR inhibitor activating autophagy, can reverse upregulation effect of p-mTOR and mTOR expression in GSCs induced by combined application of TMZ plus nicardipine (Figures 5D, 2G). Rapamycin-treated GSCs showed decline of p62 expression and accumulation of LC3-II (Figure 5A–5C, 5E, 5F), indicating that rapamycin can promote GSCs autophagy and reverse autophagy inhibition of GSCs induced by combined application of nicardipine with TMZ. Flow cytometry was carried out for further exploring the interaction between apoptosis induced by combined treatment and autophagy, which revealed that rapamycin weakened GSCs apoptosis induced by combined treatment (Figure 6A–6D). Western blot analysis on apoptotic-related proteins suggested that rapamycin can reduce Bax/Bcl-2 ratio in GSCs treated with TMZ and nicardipine (Figure 6E–6G). Collectively, these findings indicated that rapamycin can decrease apoptotic efficiency in GSCs treated with TMZ and nicardipine via promoting GSCs autophagy.

### Nicardipine can sensitize GSCs inhibition effect of TMZ *in vivo*

Sensitization effect of nicardipine on temozolomide against GSCs *in vivo* was also evaluated. There was no obvious drug-related side effect during combined treatment in the tumor bearing mice. The OS of tumor bearing mice was recorded. The median survival of the normal saline control group was 29 days, the temozolomide group was 38 days, and the combined treatment group was 45 days. Kaplan Meier survival curve analysis showed that the median survival of the combined treatment group was longer than that of the other two groups (*p*<0.05) (Figure 6H).

## DISCUSSION

TMZ has been widely applied in the standard chemotherapy against malignant gliomas. However, recent clinical practice showed that TMZ was less effective for prolonging patient survival in a considerable number of high grades glioma patients with high MGMT expression without promoter region methylation. Therefore, screening novel potential drugs or therapeutic strategies that help to enhance the bioavailability of TMZ and overcome chemoresistance are under urgent need. Previous studies have demonstrated that high resistance to autophagic cell death and apoptosis was partially responsible for glioma resistance to TMZ [4].

The current studies showed that nicardipine, a common calcium channel blocker, can improve antitumor effects of TMZ by promoting autophagy and apoptosis of GSCs, indicating that nicardipine can sensitize GSCs to TMZ-induced cytotoxicity. Nicardipine, a hepatically metabolized dihydropyridine-type calcium channel blocker with high lipid solubility and low dissociation constant, can pass through blood-brain barrier [27–29] and has been proposed to function as an adjuvant for chemotherapeutic or other drugs. It has been reported that nicardipine can decrease U251 cell proliferation, and completely block the growth-stimulating effects of epidermal growth factor (EGF) [30]. In addition, nicardipine had synergistic effect with chemotherapeutic drugs. Our previous studies reported that nicardipine enhanced mitoxantrone-induced cytotoxicity against glioma through competitive inhibition of ABCG2, confirming the sensitizing role of nicardipine with chemotherapeutic agent on gliomas [31]. However, the molecular mechanisms and the regulatory pathways which was involved in synergistic effect of nicardipine with TMZ against GSCs have not been fully elucidated. Our results suggest that nicardipine, acting as an autophagy inhibitor, can sensitize GSCs to TMZ through inhibiting autophagy, for autophagy was considered a protective response in glioma cells during chemotherapy.

Dysregulation of cell death programs, such as autophagy and apoptosis [32] was considered a major contributor to chemo- and radioresistance of GBM. The role of autophagy in cancer can be either prosurvival or prodeath, depending on the specific biological or experimental context in different types of tumor cells [20, 33]. Under stress conditions such as hypoxia and starvation, autophagy provides convenient nutrients and energy for cell metabolism, contributing to tumor progression [34]. On the contrary, under stimulation by chemotherapeutic agents or radiotherapy, autophagy may act as a tumor suppressor by degrading damaged organelles, protein aggregates, and oxidized products, thus leading to tumor cell death or apoptosis [35]. It has been demonstrated that rapamycin, an autophagy activator, can promote glioma cell death and sensitize glioma cells to TMZ treatment by upregulating the expression of Beclin-1, ATG5, and LC3-II [36], suggesting a possible adjuvant role of autophagy on enhancing the response to conventional chemotherapy against gliomas. The current studies showed that autophagy inhibition and apoptosis increasing can be achieved in GSCs after treatment with both TMZ and nicardipine against GSCs, which can be reversed by rapamycin, an inhibitor of mTOR, leading to apoptosis decreasing and autophagy increasing.

Notably, the interaction between the two biological processes autophagy and apoptosis was complex, especially in the sophisticated cancer development processes, and treatment-induced tumor progression. Our results suggest that combination of TMZ and nicardipine affected both autophagy and apoptosis of GSCs leading to overcoming natural resistance of GSCs to TMZ, which deserved further clinical investigations.

## MATERIALS AND METHODS

### Cell culture and materials

The primary cultured GSCs SU4 and SU5 were derived from fresh surgical specimen of human GBM tissues using a method described previously with informed consent [37]. The study was approved by the Ethics Committee of the Second Affiliated Hospital of Soochow University. Cell lines were maintained in Dulbecco’s modified Eagle’s medium/F12 (Gibco, Grand Island, NY, USA) supplemented with B-27 (Gibco, Grand Island, NY, USA), 20 ng/mL recombinant human epidermal growth factor (EGF; Gibco, Grand Island, NY, USA), 20 ng/mL basic fibroblast growth factor (bFGF; Gibco, Grand Island, NY, USA) and antibiotics (100 U/mL penicillin and 100 mg/L streptomycin (HyClone, South Logan, UT, USA)).

TMZ, Nicardipine, chloroquine (CQ), rapamycin, laminine and poly-L-ornithine solutions were purchased from Sigma-Aldrich Co. (St. Louis, MO, USA). Dimethyl sulfoxide was the product of MP Biomedicals, LLC (Santa Ana, CA, USA). Antibodies against β-actin, Bcl-2, Bax, p62, LC3B, AKT, p-AKT, mTOR and p-mTOR, were purchased from Cell Signaling Technology (Danvers, MA, USA). The secondary biotinylated antibodies were from Jackson ImmunoResearch Laboratories (West Grove, PA, USA).

### Neurosphere formation and immunofluorescence staining

GSCs were seeded in 96-well plates (2×10^3^ cells per well) and supplemented with 50 μL culture medium every 3 days. Then neurospheres formation was observed under a microscope (Nikon, Tokyo, Japan) after 10-14 days of incubation. Immunofluorescence staining of CD133, CD44, Nestin was carried out as described [38].

### mCherry- GFP- LC3 lentivirus transfection

mCherry- GFP -LC3 lentivirus was applied to transfect glioma stem cells SU4 and SU5 to detect autophagy flux, following the manufacturer’s instructions. Then the GSCs were treated with TMZ (400μM), nicardipine (20μM), or both, respectively, for 48 h, and intracellular fluorescence was observed by confocal microscopy, yellow fluorescence indicated the presence of autophagosomes, and red fluorescence suggested the existence of autolysosomes.

### Cell survival assay

GSCs were cultured in a 96-well plate (2×10^3^ cells per well) and incubated at 37° C for 12 h, then were treated with different dosage of TMZ, the final concentration of TMZ applied was as following, 0, 50μM, 100μM, 200μM, 400μM, 800μM, 1600μM. Nicardipine was also administered at a series of final concentration gradient, 0, 5μM, 10μM, 20μM, 40μM, 60μM, 80μM. Cells were treated with TMZ, nicardipine, and both of the above mentioned drugs, respectively. CCK-8 solution (Dojindo, Kumamoto, Japan) was supplemented into each well, incubated for 2 h at 37° C on a shaker. Subsequently, the absorbance values were measured at 490 nm on a microplate reader EL×800 (BioTek, Winooski, Vermont, USA). The viability of untreated cells was set as 100%. The experiment was repeated three times.

### Cell apoptosis assay

GSCs were seeded in 6-well plate (2×10^5^ cells per well) and treated with TMZ (400μM), nicardipine (20μM) or both, with or without rapamycin (50 nM), incubated for 48 h. A total of 1×10^5^ GSCs were resuspended in the binding buffer and stained with 5 μL of Annexin V-fluorescein isothiocyanate (FITC) and 5 μL of propidium iodide (PI; BD Pharmingen, San Diego, CA, USA) for 30 min, then were detected by flow cytometry (Guava EasyCyte 6HT-2L, Merck Millipore, Darmstadt, Germany). The early apoptotic cells located in the lower right quadrant were calculated as the percentage of apoptotic cells. The experiment was repeated three times.

### Western blot

Cells were cultured in 6-well plate and treated with TMZ (400μM), Nic (20μM)/CQ (10μM) or both, with or without rapamycin (50 nM) for 48 h, then cells were digested with accutase and washed thoroughly with PBS for three times, followed by lysing cells in RIPA buffer (Beyotime, Shanghai, China) supplemented with 1% protease and phosphatase inhibitor cocktail (Roche, Basel, Switzerland). After centrifugation at 12000 g for 2 min at 4° C, protein concentration was determined with a standard BCA Protein Assay Kit (Thermo Fisher Scientific, Waltham, MA, USA). Western blot was performed according to the standard protocol. The mitochondrial protein was extracted with a mitochondrial isolation kit (Beyotime Biotechnology; Jiangsu, China), according to the manufacturer’s instructions. Primary antibodies against Bcl-2 (#2872), Bax (#2772), p62 (#8025), LC3 (#3868), mTOR (#2983), p-mTOR (#5536), AKT (#4691) and p-AKT (#4060) were purchased from Cell Signaling Technology (Danvers, MA, USA). Rabbit anti-β-actin (#4970) was used as normal loading control while rabbit anti-CoxIV (#4850) was applied as mitochondrial loading control. An ECL plus kit (Thermo Fisher Scientific, Waltham, MA, USA) and a ChemiDoc Touch Imaging System (BioRad, Hercules, CA, USA) were applied to detect the chemiluminescence signals. Each experiment was repeated three times.

### Evaluation of nicardipine sensitizing TMZ against GSCs in orthotopic GSCs model

10 μl cell suspension containing 10^6^ GSCs was implanted into the right caudate nucleus of 4-week-old BALB / C nude mice with stereotactic techniques (nude mice were obtained from the Nanjing Institute of Model Animals). After 7 days, the mice were randomly divided into three groups. TMZ group (n=5): intragastric administration of TMZ (50 mg / kg, once a day for 5 days). TMZ + nicardipine group (n=5): TMZ was administration according to the above method, and nicardipine (1.5mg/kg) was injected intraperitoneally slowly on the same day. Control group (n=5): intraperitoneal injection of equal volume of normal saline. The overall survival (OS) time of mice was recorded and analyzed by Kaplan-Meier survival curves.

### Statistical analysis

All data was collected and analyzed with GraphPad Prism 7 (GraphPad Software), and expressed as mean ± SD values. Student’s t-test (two tailed) or one-way ANOVA was used for statistical analysis. *p* value < 0.05 was considered to reflect a statistically significant difference.

### Ethics approval and consent to participate

Procedures performed in the study were in accordance with the ethical standards of the institutional research committee.

### Statement of human and animal rights

The study was approved by the Ethics Committee of the Second Affiliated Hospital of Soochow University.

### Availability of data and materials

The datasets are available from the corresponding author on reasonable request.

## Supplementary Material

Supplementary Figure 1
