# Supplementary material for: Nicardipine sensitizes temozolomide by inhibiting autophagy and promoting cell apoptosis in glioma stem cells
Source: Aging (Albany NY). 2021 Feb 17;13(5):6820–31. doi: 10.18632/aging.202539 (PMC7993688; doi:10.18632/aging.202539)
Supplement: Supplementary Figure 1 [file aging-13-202539-s001.pdf]

SUPPLEMENTARY FIGURE

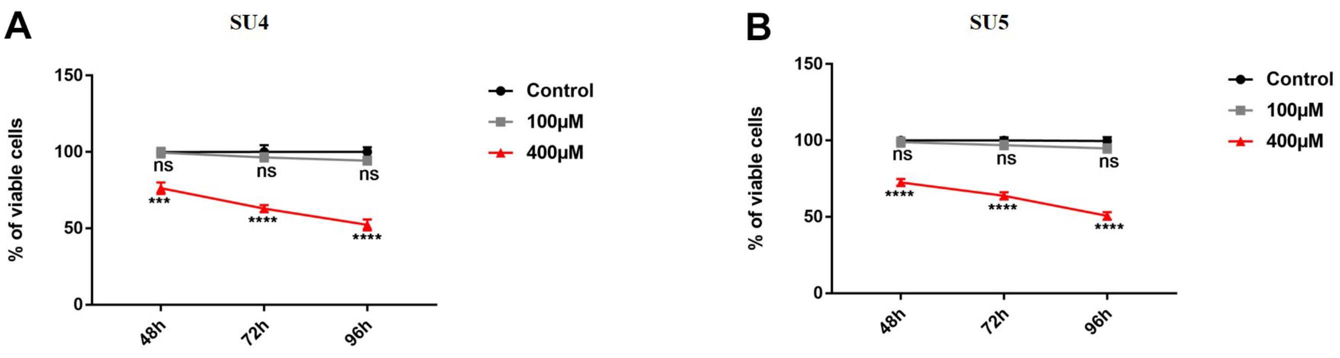

Supplemental Figure 1. The viability of GSCs in different concentration and time point of TMZ. Cell viability of GSCs SU4 and SU5 determined by CCK-8 assay after 48 h, 72 h or 96 h of TMZ (100 μM or 400 μM) treatment (**A**, **B**). *** $p < 0.001$, **** $p < 0.0001$.
